# Author Correction: Outflows from the youngest stars are mostly molecular

**DOI:** 10.1038/s41586-023-06744-8

**Published:** 2023-10-16

**Authors:** T. P. Ray, M. J. McCaughrean, A. Caratti o Garatti, P. J. Kavanagh, K. Justtanont, E. F. van Dishoeck, M. Reitsma, H. Beuther, L. Francis, C. Gieser, P. Klaassen, G. Perotti, L. Tychoniec, M. van Gelder, L. Colina, Th. R. Greve, M. Güdel, Th. Henning, P. O. Lagage, G. Östlin, B. Vandenbussche, C. Waelkens, G. Wright

**Affiliations:** 1 Dublin Institute for Advanced Studies, Dublin, Ireland; 2 School of Physics, Trinity College Dublin, Dublin, Ireland; 3 European Space Agency, ESTEC, Noordwijk, the Netherlands; 4 INAF - Osservatorio Astronomico di Capodimonte, Naples, Italy; 5 Department of Experimental Physics, Maynooth University, Maynooth, Ireland; 6 Department of Space, Earth and Environment, Chalmers University of Technology, Onsala Space Observatory, Onsala, Sweden; 7 Leiden Observatory, Leiden University, Leiden, the Netherlands; 8 Max-Planck-Institut für Astronomie (MPIA), Heidelberg, Germany; 9 Max-Planck-Institut für Extraterrestrische Physik, Garching, Germany; 10 UK Astronomy Technology Centre, Royal Observatory Edinburgh, Edinburgh, UK; 11 European Southern Observatory, Garching, Germany; 12 Centro de Astrobiología (CAB, CSIC-INTA), Carretera de Ajalvir, Torrejón de Ardoz, Spain; 13 DTU Space, Technical University of Denmark, Kongens Lyngby, Denmark; 14 Department of Astrophysics, University of Vienna, Vienna, Austria; 15 ETH Zürich, Institute for Particle Physics and Astrophysics, Zurich, Switzerland; 16 Université Paris-Saclay, Université Paris Cité, CEA, CNRS, AIM, Gif-sur-Yvette, France; 17 Department of Astronomy, Stockholm University, AlbaNova University Center, Stockholm, Sweden; 18 Institute of Astronomy, KU Leuven, Leuven, Belgium

**Keywords:** Astronomy and astrophysics, Space physics

Correction to: *Nature* 10.1038/s41586-023-06551-1 Published online 24 August 2023

In the version of the article initially published, there was an error in the tenth paragraph, where in the sentence now reading “… where CO flux dominates (positive; light yellow-white) and, conversely, where H2 emission prevails (negative; dark orange-red)”, the colours “light yellow-white” and “dark orange-red” were inadvertently swapped. This has been corrected in the HTML and PDF versions of the article.

